# Essential fatty acid deficiency in parenteral nutrition: Historical perspective and modern solutions, a narrative review

**DOI:** 10.1002/ncp.11278

**Published:** 2025-02-17

**Authors:** Jodi Wolff, Mary Petrea Cober, Katie A. Huff

**Affiliations:** ^1^ Baxter Healthcare Corporation Deerfield Illinois USA; ^2^ College of Pharmacy Northeast Ohio Medical University Rootstown Ohio USA; ^3^ Division of Neonatal‐Perinatal Medicine, Department of Pediatrics Indiana University School of Medicine Indianapolis Indiana USA

**Keywords:** essential fatty acid, essential fatty acid deficiency, intravenous lipid emulsion, lipid, lipid injectable emulsion

## Abstract

Essential fatty acid deficiency (EFAD) may occur in the setting of inadequate fat intake, malabsorption, malnutrition, and altered fat metabolism. Humans lack the enzymes to synthesize the essential acids linoleic acid and alpha‐linolenic acid, so they must be obtained from the diet. Patients dependent on parenteral nutrition need adequate amounts of these essential fatty acids supplied in lipid injectable emulsions (ILEs). With the increasing use of multicomponent ILEs that are lower in linoleic and alpha‐linolenic acid, it is imperative that clinicians understand appropriate dosing to prevent EFAD. An understanding of fatty acid composition and metabolic pathways is important, as the use of the Holman Index (triene:tetraene ratio) alone may lead to an inaccurate diagnosis of EFAD.

## INTRODUCTION

Two polyunsaturated fatty acids, linoleic acid and alpha‐linolenic acid, are considered essential in humans because they cannot be synthesized within the body. The need for these fatty acids in the human diet was first described in the early 20th century.^1^ However, even after the discovery of these fatty acids, it took decades to fully understand the complications that can result when there is true essential fatty acid deficiency (EFAD). Holman first described the use of the triene (Mead acid) to tetraene (arachidonic acid [ARA]) ratio (T:T ratio) to identify EFAD, which he classified as 0.4.^2^ Decades later, the Holman index threshold of 0.2 is commonly used to diagnose EFAD based on further data from Holman et al in healthy controls that established reference values at the time.^3^ This is despite the availability of more recent fatty acid panels with reference values in healthy populations with a lower reported T:T ratio.^4^, ^5^ With the introduction of newer lipid injectable emulsions (ILEs) with variable oil and fatty acid content, alterations in fatty acid levels obtained from patients receiving these ILEs reflect the ILE fatty acid composition and make identifying EFAD more complex. With this innovation in ILEs, the T:T ratio alone is felt to be insufficient to understand and describe a patient's fatty acid status. The risk for EFAD has also increased in high‐risk patient populations as the newer ILEs have lower essential fatty acid content compared with the original soybean ILEs. As a result, knowledge regarding dosing is needed to prevent EFAD. The goal of this review is to describe the historic context of EFAD and relate this foundation to the usage of currently available ILE products in various patient populations from the preterm neonate to the adult.

## HISTORY OF EFAD

Before 1929, the need for fatty acids within one's diet was not considered essential. In 1929, George Oswald Burr determined the need for specific dietary fatty acids to prevent deficiency, which was observed in rats fed a fat‐free diet.^6^ Subsequently, he discovered the provision of a small amount of linoleic acid, an 18‐carbon ω‐6 polyunsaturated fatty acid with two double bonds, could reverse this observed deficiency.^6^ However, questions arose as to whether linoleic acid was an essential component for the human diet, given the lack of a similar deficiency being observed in humans.^1^ To determine the essential nature of linoleic acid in the human diet, Burr, along with his student Adril Hansen, studied a healthy adult male receiving a nearly fat‐free diet for 6 months in whom they observed a 40% reduction in serum linoleic acid and ARA.^1^ This reduction was comparable to that observed in rats who received a similar diet but without the same clinical symptoms of deficiency, except for gradual weight loss. Burr and his colleagues proposed the same clinical signs and symptoms would be the result, however, if the fat‐free diet continued. In addition, Burr and colleagues also described the action of alpha‐linolenic acid, first noting in 1932 that alpha‐linolenic acid supplied to rats fed fat‐free diets partially improved growth.^7^ This supplementation with alpha‐linolenic acid, however, made linoleic acid less effective, raising questions about its effectiveness and true essentiality. It was not until the importance of the alpha‐linolenic acid metabolites eicosapentaenoic acid (EPA) and docosahexaenoic acid (DHA) were defined that alpha‐linolenic acid was felt to be an essential fatty acid.^8^ The first reported deficiency of alpha‐linolenic acid in humans was described in 1982 when a 6‐year‐old child receiving parenteral nutrition (PN) containing an ILE composed of safflower oil (Liposyn I, Abbott Laboratories) rich in only linoleic acid developed clinical symptoms.^9^, ^10^ The resulting signs and symptoms of alpha‐linolenic acid deficiency included numbness, paresthesia, weakness, inability to walk, pain in the legs, and blurred vision, which resolved within 3 months of increasing the alpha‐linoleic acid content provided within the daily ILE. The authors concluded that ω‐3 polyunsaturated fatty acids, such as alpha‐linolenic acid, are the essential dietary components to provide normal nerve function in the human body.^8^, ^11^

Because observable EFAD was excessively rare in the human population, many scientists felt the deficiency was of little concern in humans. However, in 1958, evidence of skin abnormalities in infants fed a low‐fat diet brought this concern again to the forefront.^12^ Of 24 infants observed on a low‐fat milk formula for at least 1 month, 15 developed skin changes consisting of dryness, leathery thickening, and some desquamation, as well as exudation.^12^ When linoleic acid was added to the diet at 2% of the total energy content, these skin abnormalities resolved, as did other adverse effects such as diarrhea and perianal irritation. The results of this study led researchers to believe infants were susceptible to EFAD and could develop it much quicker than older humans.^13^ Further studies in this population concluded that a minimum of 1% of a healthy infant's energy content as linoleic acid was necessary to prevent EFAD.^14^ In a large study evaluating the role of linoleic acid in infants (premature 109, full term 319), participants were randomized to one of five diets with linoleic acid content ranging from 0.04% to 7.3% of total energy.^15^ Infants receiving 0.04% and 0.07% developed clinical EFAD. When linoleic acid was given as ≥1% of total energy, the clinical manifestations of EFAD resolved. For improved energy efficiency and usage, a higher percentage of 4% linoleic acid has been recommended. This recommendation is based on decreased energy needs to maintain growth in infants with at least 4% of total energy from linoleic acid, the intake of breastfed infants.^14^, ^16^

With the advent of long‐term PN in the late 1960s and early 1970s devoid of lipid, EFAD was observed in adult patients receiving PN.^8^ The lack of lipid provision was primarily limited to the US, as the first stable ILE, Lipomul, manufactured by the Upjohn Company and consisting of 15% cotton seed oil, was removed from the market because of serious adverse effects.^17^, ^18^ The adverse effects observed included chills, fever, nausea, vomiting, dyspnea, hypoxia, and hypotension. Given the severity of these observed adverse events, the development and use of other ILEs in the US was severely hindered until the late 1970s when a soybean oil–based ILE (SO‐ILE), Intralipid (Fresenius Kabi), was approved by the US Food and Drug Administration (FDA). Researchers determined why adults dependent on PN were less likely to experience EFAD than infants dependent on PN. Two experiments in healthy adult males determined that the continuous provision of fat‐free nutrition, either via the intravenous or nasogastric route, resulted in EFAD.^19^ By studying biochemical changes in one of the study participant's fatty acid profile, it was noted that evidence of a partial repletion of the necessary essential fatty acids was observed with cyclic administration of the same nutrition. Wene and colleagues proposed that essential fatty acids could be repleted because of the lipolysis that occurred during the periods of time in which the patient did not receive continuous glucose.^19^ A study evaluating the amount of linoleic acid necessary to prevent EFAD in 97 men receiving continuous PN for a minimum of 14 days found that 3.2% of the energy given as intravenous fat and 7.7 g of linoleic acid given via a 10% SO‐ILE prevented EFAD.^20^ As linoleic acid within the body's fat stores diminishes, the likelihood of EFAD increases. This likely explained the relatively quick development of EFAD in infants with their low stores of linoleic acid as opposed to a well‐nourished healthy adult male and the greater risk of EFAD among malnourished adults as time receiving PN continued.^21^ With the inclusion of ILEs primarily consisting of soybean oil rich in linoleic acid as a standard component of PN, the incidence of EFAD decreased. However, with the introduction of newer composite ILEs with decreased proportions of soybean oil, there is an increase in the possibility of seeing EFAD.

## BIOCHEMISTRY

**Figure 1:**
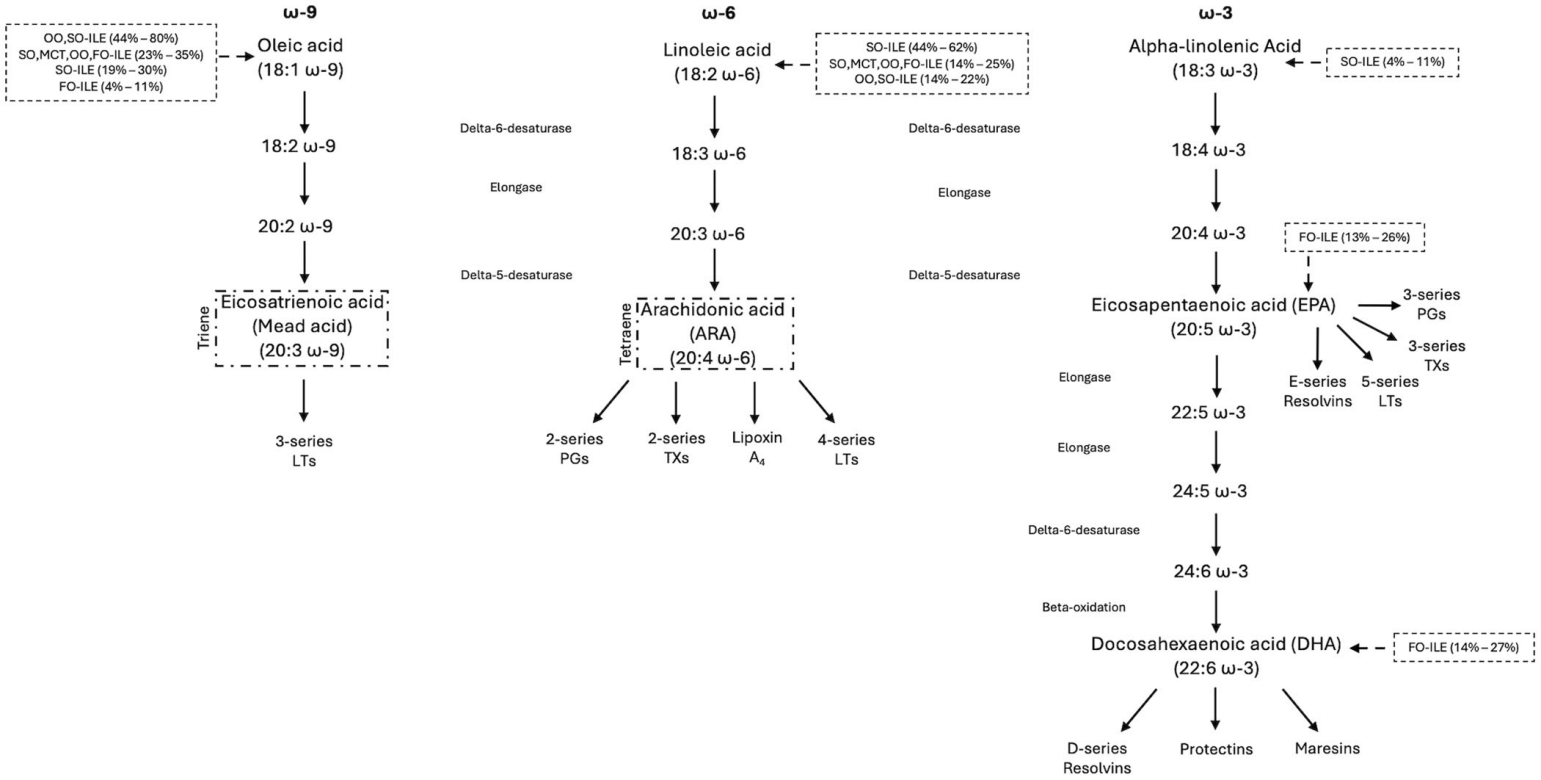
Schematic of essential fatty acid metabolism in humans including downstream metabolites and ILE sources. All pathways share the same enzymes with these enzymes having a preference for ω‐3 > ω‐6 > ω‐9 fatty acids. For calculating the triene:tetraene ratio for essential fatty acid deficiency diagnosis, Mead acid (triene) and arachidonic acid (tetraene) are highlighted. Figure drafted using data from multiple sources.^22^, ^23^, ^24^, ^25^, ^26^, ^27^, ^28^, ^29^, ^30^ FO, fish oil; ILE, lipid injectable emulsion; LT, leukotriene; MCT, medium‐chain triglyceride; OO, olive oil; PG, prostaglandin; SO, soybean oil; TX, thromboxane.

Two fatty acids (the ω‐3 fatty acid alpha‐linolenic acid and ω‐6 fatty acid linoleic acid) are considered essential in the human body, as they cannot be synthesized, given the lack of the desaturase enzymes necessary to insert a double bond at the ω‐3 and ω‐6 positions.^22^ These fatty acids undergo conversion through desaturation and elongation to other important fatty acids including ARA, EPA, and DHA (Figure 1).^22^, ^23^, ^24^, ^25^, ^26^, ^27^, ^28^, ^29^, ^30^ In preterm infants and those with severe liver disease, DHA and ARA are considered conditionally essential fatty acids, as the enzymes needed for their formation have decreased activity.^31^ The synthesis of downstream fatty acids is influenced by the relative intakes of other fatty acids, given that ω‐3, 6, and 9 fatty acids share desaturase and elongase enzymes. These enzymes have the greatest affinity for ω‐3 fatty acids, followed by ω‐6 and then ω‐9 fatty acids.^31^

Most fatty acids found in humans can be synthesized in the body. The dietary fatty acids are incorporated into triglycerides (triacyclglycerols), phospholipids, and other complex lipids.^32^ These lipids are incorporated into cell membranes and tissues, influencing cell function and signaling, including gene expression via influences on transcription factors.^32^ On a larger scale, fatty acids influence tissue and organ composition and function. For example, linoleic acid is highly incorporated into ceramides within the skin, helping to influence its barrier function and ability to prevent water loss.^32^ ARA and DHA are highly concentrated within the developing brain and retina.^33^ Additionally, EPA and DHA have been shown to influence brain function in childhood and beyond, including behavioral, mood, and psychiatric disorders.^32^ In individuals who are parenterally supported, the choice of ILE and its oil composition influences the fatty acid levels directly and can ultimately influence body functions.^34^ Additional actions of the polyunsaturated fatty acids include their downstream metabolites, eicosanoids (eg, prostaglandins [PGs], thromboxane, and leukotrienes), and proresolving mediators (eg, resolvins, protectins, and maresins), some of which are depicted in Figure 1.^25^, ^32^ Historically, fatty acids and their downstream metabolites have been categorized as proinflammatory or anti‐inflammatory. Newer information has shown some metabolites can have dual functions.^22^ For example, PGE‐2 is derived from ARA and has been associated with increases in interleukin‐6 production, influencing the degree of inflammatory response to a stimulus, and was historically labeled as “proinflammatory.” However, PGE‐2 has more recently been shown to inhibit production of proinflammatory cytokines interleukin‐1 and tumor necrosis factor alpha, having an anti‐inflammatory effect at times.^22^

## RISK FACTORS FOR EFAD

Any patient with a limited intake of linoleic acid is at risk for EFAD. The nutrition status (eg, healthy or nourished vs malnourished) and age of the patient (eg, adult, infant, or preterm neonate) as well as the means of energy provision (eg, continuous glucose vs cyclic) determine the time to develop EFAD. Those at risk for EFAD include any patient receiving an intake of either PN or enteral nutrition with insufficient linoleic acid, those with malabsorption states (eg, cystic fibrosis, pancreatic insufficiency, or postbariatric surgery), and those who lack linoleic acid–containing adipose stores (eg, infants, especially premature neonates, or the malnourished).^35^ In addition to these commonly recognized groups, one must consider patients who, owing to unforeseen circumstances, are unable to achieve sufficient infusion of ILE. These groups include those impacted by ILE shortages, those receiving ILE minimization to treat intestinal failure–associated liver disease (IFALD), those unable to receive either in part or completely the ordered daily ILE dose owing to lack of intravenous access or intravenous solution incompatibility, and those with potential allergies to components of ILEs. A final group of at‐risk patients is related more to a lack of appropriate prescriber knowledge and includes those who are fungemic who are not receiving ILEs because of historic concerns for the receipt of ILEs and fungemia, an approach that has been proven unnecessary,^36^ and those who are incorrectly dosed. This last group is of greatest concern currently given reduced linoleic acid–containing soybean oil in newer composite ILE formulations.

## OVERVIEW OF ILEs

### SO‐ILEs

SO‐ILEs, such as Intralipid and Nutrilipid (B. Braun) have been available in the US since 1975 and 1993, respectively.^26^, ^27^ These 100% SO‐ILEs contain considerable amounts of ω‐6 polyunsaturated fatty acids rich in linoleic acid (50%) in addition to alpha‐linolenic acid (9%) (Table 1).^37^ Given the linoleic and alpha‐linolenic content, the likelihood of EFAD with SO‐ILE is low if dosed appropriately. However, soybean oil–sparing ILE regimens may increase the risk of EFAD.^38^ The high phytosterol content of SO‐ILEs may have inflammatory and hepatoxic effects.^39^, ^40^ Additionally, SO‐ILE is low in vitamin E, an antioxidant substrate that is necessary to minimize lipid peroxidation. The vitamin E present is in the form of gamma‐tocopherol, which has lower biological activity than the alpha‐tocopherol present in fish oil.^41^ SO‐ILE has been associated with the development of IFALD because of increased inflammation and impaired biliary flow.^39^ IFALD is defined as liver injury (cholestasis, steatosis, and fibrosis) related to intestinal failure and associated PN therapy in the absence of other causes of liver disease.^42^, ^43^ Some research has shown that restricting SO‐ILE to ≤1 g/kg/day can improve liver function tests and decrease the incidence of IFALD.^38^, ^44^

**Table 1:** Lipid emulsions available in the United States with oil composition and fatty acid content.

	OO,SO‐ILE 20%^30^	SO‐ILE 20%^26^, ^27^	SO,MCT,OO,FO‐ILE 20%^29^	FO‐ILE 10%^28^
Oil g/100 ml (%)				
Olive	16 (80)	0	5 (25)	0
Soybean	4 (20)	20 (100)	6 (30)	0
MCT	0	0	6 (30)	0
Fish oil	0	0	3 (15)	10 (100)
Components (g/100 ml)				
Phospholipids	1.2	1.2	1.2	1.2
Glycerol/glycerin	2.25	2.25–2.5	2.5	2.5
Sodium oleate	0.03	0.03	0.03	–
Fatty acid composition, %				
MUFAs, %				
Oleic acid	44.3–79.5	17–30	23–35	4–11
Palmitoleic	0.0–3.2	–	–	4–10
PUFAs, %				
Linoleic acid, %	13.8–22.0	44–62	14–25	1.5
Alpha‐linolenic acid, %	0.5–4.2	4–11	1.5–3.5	1.1
Arachidonic acid, %	0.16	0	0.27	0.2–2
EPA, %	0	0	1–3.5	13–26
DHA, %	0	0	1–3.5	14–27
SFAs, %				
Palmitic acid	7.6–19.3	7–14	7–12	4–12
Stearic acid	0.7–5.0	1.4–5.5	1.5–4	–
MCFAs, %				
Capric acid	–	–	5–15	–
Caprylic acid	–	–	13–24	–

*Note*: Data adapted using sources noted in the table as well as additional manufacturer data.

Abbreviations: DHA, docosahexaenoic acid; EPA, eicosapentaenoic acid; FO, fish oil; ILE, lipid injectable emulsion; MCFA, medium‐chain fatty acid; MCT, medium‐chain triglyceride; MUFA, monounsaturated fatty acid; OO, olive oil; PUFA, polyunsaturated fatty acid; SFA, saturated fatty acid; SO, soybean oil.

European Society for Clinical Nutrition and Metabolism (ESPEN) guidelines for adult patients receiving long‐term PN recommend limiting SO‐ILE to ≤1 g/kg/day, as higher doses have been associated with a significant increase of IFALD.^43^ European Society for Pediatric Gastroenterology Hepatology and Nutrition (ESPGHAN) guidelines state pediatric patients requiring more than a few days of PN should no longer receive 100% SO‐ILE and should transition to composite ILEs with or without fish oil.^45^ They also recommend pediatric patients with IFALD have ILE dosing altered by stopping SO‐ILE, decreasing the ILE dose, or replacing it with fish oil.^45^ A recent American Society for Parenteral and Enteral Nutrition (ASPEN) guideline based on a systematic review of the literature from 2001 to 2023 in nonsurgical infants <37 weeks gestational age concluded that no evidence exists for the reduction of IFALD risk with any specific ILE formulation, including 100% SO‐ILE and composite multioil ILEs with or without fish oil.^46^ This was consistent with systematic reviews that evaluated the impact of ILE formulation on clinical outcomes such as IFALD, bronchopulmonary dysplasia, retinopathy of prematurity, growth, and death in preterm and late preterm infants. These reviews concluded insufficient evidence to determine ILE‐specific clinical benefits.^47^, ^48^

### Soybean, medium‐chain triglyceride, olive oil, and fish oil–based ILE (SO,MCT,OO,FO‐ILE)

The FDA approved SMOFlipid (Fresenius Kabi) in the US in 2016 for adult use and in 2022 for use in pediatrics, but it has been used outside the US since the late 1990s.^40^ SMOFlipid consists of 30% soybean oil, 30% medium‐chain triglyceride, 25% olive oil, and 15% fish oil (Table 1). The fish oil component provides a source of ω‐3 polyunsaturated fatty acids with anti‐inflammatory properties. The fatty acid content includes 21.4% linoleic acid and 2.5% alpha‐linolenic acid.^37^ Goulet and colleagues evaluated the safety and efficacy of SO,MCT,OO,FO‐ILE in children receiving home PN at least 1 year and receiving SO,MCT,OO,FO‐ILE at least 6 months with a median age of 6 years.^49^ Serial samples in a portion of the patients aged >2 years receiving SO,MCT,OO,FO‐ILE with a median dose of 2 g/kg/day compared with controls not receiving PN showed no difference in T:T ratio but did show higher EPA and DHA and lower ARA in those receiving SO,MCT,OO,FO‐ILE. The groups had similar growth, and the control group had lower conjugated bilirubin levels. From this, the authors concluded that SO,MCT,OO,FO‐ILE is safe for long‐term use in pediatric patients. Other studies with infants receiving SO,MCT,OO,FO‐ILE at a dose of ≤2 g/kg/day have reported elevated T:T ratios.^50^, ^51^

A multicenter, prospective randomized controlled study to evaluate the safety of SO,MCT,OO,FO‐ILE compared with SO‐ILE in hospitalized neonates and infants with gastrointestinal diagnoses expected to require PN and ILE for 28 days was conducted.^52^ Infants were ≥24 weeks postmenstrual age with a birth weight ≥750 g. The primary end point was the number of patients with a conjugated bilirubin >2.0 mg/dl in each ILE group. Secondary objectives included overall safety of SO,MCT,OO,FO‐ILE including the rate of EFAD, defined by a T:T ratio >0.2. There was no significant difference in the risk of having an elevated conjugated bilirubin when comparing SO‐ILE (3.8%) with SO,MCT,OO,FO‐ILE (2.4%) (risk ratio 0.59 [95% CI, 0.09–3.76]) and none of the patients in either group developed EFAD.^52^ Of note, because of a lower rate of elevated conjugated bilirubin than expected in the control group, a smaller sample size of patients was enrolled than deemed necessary to detect a true difference in cholestasis.

In adults, ASPEN notes the potential uses of SO,MCT,OO,FO‐ILE including in patients receiving long‐term PN, in those with an inflammatory state or in those with elevated liver function test (LFT) results or triglycerides.^53^ A single‐center experience of SO,MCT,OO,FO‐ILE in adult patients receiving home PN for ≥12 months found SO,MCT,OO,FO‐ILE was well tolerated.^54^ They also noted an ability to increase overall fat‐energy delivery while decreasing carbohydrate energy, with improvement in LFT results, bilirubin levels, and alpha‐tocopherol levels in patients. A recent network meta‐analysis compared and ranked various ILEs with and without fish oil and suggested fish oil–enriched PN may improve clinical outcomes in hospitalized adult patients.^55^ A significant reduction of infection risk, sepsis, and length of stay in the fish oil–enriched PN vs standard PN was observed. It is important to note that all fish oil–containing ILEs were grouped together into the fish oil–enriched PN group, and the analysis did not separate ILE types (ie, 100% FO‐ILE, 15% fish oil, and 10% fish oil), which is important because clinical effects may be dose dependent.

The report from the International Lipids in PN Summit 2022 stated there is sufficient evidence for use of fish oil–containing ILEs in critically ill adults, including surgical and nonsurgical patients.^56^, ^57^ This panel of experts recommended fish oil–containing lipid emulsions in critically ill adults as part of PN even in the first week of use.^56^ The rationale for this recommendation is that ω‐3 polyunsaturated fatty acids, including EPA and DHA, are precursors to the specialized proresolving mediators (Figure 1) that may promote inflammation resolution, tissue repair, and preservation of skeletal mass.^58^ The group also concluded that when given as recommended, SO,MCT,OO,FO‐ILE and fish oil–based ILEs do not lead to EFAD in clinical practice.

### Olive oil–based ILE

Olive oil–based ILEs have been used in Europe in both pediatric and adult patients since the 1990s. In the US, the olive oil– and soybean oil–based ILE (OO,SO‐ILE) Clinolipid (Baxter) has been used in adults since 2019.^30^ OO,SO‐ILE obtained an indication for use in premature infants and pediatric patients in 2024. OO,SO‐ILE is 80% olive oil and 20% soybean oil with a linoleic acid content of 18% and alpha‐linolenic content of 2% (Table 1).^37^ It is high in oleic acid, a monounsaturated ω‐9 fatty acid that is less prone to lipid peroxidation. It contains the naturally occurring form of vitamin E, alpha‐tocopherol, which is the most bioactive form and does not require the addition of synthetic alpha‐tocopherol that is added to other ILEs higher in unsaturated fat.^59^ When comparing vitamin E levels in infants receiving SO‐ILE and OO,SO‐ILE, infants receiving OO,SO‐ILE had vitamin E levels closer to that observed in breastfed neonates, likely because of the lower polyunsaturated fatty acid and increased antioxidant intake with OO,SO‐ILE.^59^ Studies have shown that when dosed appropriately, OO,SO‐ILEs can be used without evidence of EFAD in pediatric and adult patients.^60^, ^61^, ^62^, ^63^, ^64^, ^65^, ^66^ A randomized controlled study in adults comparing OO,SO‐ILE with SO,MCT,OO,FO‐ILE found that after 60 days, none of the patients developed EFAD (defined as a T:T ratio >0.2) receiving 20 g of ILE per day.^67^ Another study evaluated EFAD in adult patients after 5 years receiving OO,SO‐ILE, receiving an average of 0.6 ± 0.4 g/day, and found no cases of an elevated T:T ratio.^64^

A study in pediatric patients receiving long‐term PN compared the red blood cell polyunsaturated fatty acid levels of those receiving OO,SO‐ILE and SO,MCT,OO,FO‐ILE with a group of healthy children.^66^ Patients receiving SO,MCT,OO,FO‐ILE had significantly higher levels of EPA and DHA and significantly lower levels of ARA than healthy children and those receiving OO,SO‐ILE. The T:T ratio of both groups was <0.2 and did not differ from the healthy children. There were no differences in growth, and neither group had liver fibrosis as determined by transient liver elastography.^66^

A recent randomized controlled multicenter study was completed to evaluate the safety of OO,SO‐ILE in pediatric patients, the majority of whom were premature neonates, receiving PN and ILE (OO,SO‐ILE or SO‐ILE) for a minimum of 7 days.^68^ The primary end point was to evaluate the risk of developing EFAD (T:T ratio >0.4 with low linoleic acid and ARA). Secondary end points included risk of PN‐associated liver disease development (direct bilirubin >2 mg/dl), adequacy of growth, and safety analysis. A total of 101 patients were randomized to receive OO,SO‐ILE or SO‐ILE. None of the patients had a T:T >0.4, and one patient in the OO,SO‐ILE group had a T:T ratio slightly >0.2 with normal linoleic acid and ARA levels, not indicative of EFAD.^68^ Growth was adequate, and the safety profile was similar with both ILEs.

### Fish oil–based ILE (FO‐ILE)

FO‐ILE was approved in 2018 by the US FDA as a source of energy and essential fatty acids in pediatric patients with PN‐associated cholestasis or IFALD. Several studies in pediatric patients have demonstrated the apparent effectiveness of FO‐ILE in reversing IFALD.^69^, ^70^, ^71^, ^72^, ^73^ Omegaven (Fresenius Kabi) is a 100% FO‐ILE with 4.4% linoleic acid and 1.8% alpha‐linolenic acid (Table 1), rich in alpha‐tocopherol and low in phytosterols.^37^ Although FO‐ILE is low in both linoleic and alpha‐linolenic acids, the development of EFAD has not been observed in patients receiving FO‐ILE monotherapy.^74^, ^75^ Research suggests fish oil contains sufficient downstream fatty acids to compensate for low linoleic and alpha‐linolenic acids.^76^ With increased DHA, increased EPA levels are observed through retroconversion of DHA, resulting in the formation of EPA and docosapentaenoic acid.^77^ Animal studies have shown that ARA can be retroconverted to linoleic acid in the setting of ARA supplementation during EFAD.^74^, ^78^ This retroconversion, however, has not been observed in humans and should not be assumed as a reason for EFAD prevention with FO‐ILE usage. It has been suggested that ARA and DHA may decrease the amount of linoleic acid necessary to prevent EFAD or even be considered essential fatty acids themselves.^79^ In cases of EFAD, FO‐ILE has been shown to normalize T:T ratios.^50^, ^80^ FO‐ILE is currently approved for use in pediatric patients; however, it has been used off‐label in adult home PN patients as a treatment for IFALD. Two case series in adults cite success in using either a combination of FO‐ILE and SO,MCT,OO,FO‐ILE or FO‐ILE monotherapy to treat IFALD when not responding to SO,MCT,FO‐ILE alone.^81^, ^82^ In total, the four patients in these series showed improved liver imaging and laboratory testing, including resolution of IFALD. Of note, none of the patients developed clinical or biochemical EFAD on a variety of FO‐ILE doses, although only two of the patients had fatty acid testing performed. Additional studies are needed in the adult population to examine the optimal dose of FO‐ILE for clinical benefit.

## MULTICOMPONENT ILEs AND EFAD

With the arrival of the newer ILE formulations in the US market, some clinicians have started dosing the multicomponent ILEs (OO,SO‐ILE and SO,MCT,OO,FO‐ILE) like SO‐ILE, including during the use of lipid minimization strategies. In the pediatric population, multiple reports of EFAD have been associated with SO,MCT,OO,FO‐ILE with a dose range of 0.5–2 g/kg/day.^50^, ^51^, ^83^, ^84^ Most of these reports are in premature neonates or infants. A variety of treatment strategies were used to treat EFAD including increasing the SO,MCT,OO,FO‐ILE dose and changing to FO‐ILE.^50^, ^51^, ^83^, ^84^ It is also important to note when comparing these reports, a variety of EFAD definitions are used, with some using the more conservative EFAD thresholds of mild (T:T ratio >0.05), moderate (T:T ratio ≥0.20), and severe EFAD (T:T ratio ≥0.40) as proposed by Cober et al.^38^, ^50^, ^51^, ^83^, ^84^ These cases serve as a reminder to consider the lipid source and essential fatty acid content when dosing various ILE formulations. The recent cases of EFAD in the literature involve the use of SO,MCT,OO,FO‐ILE. This is potentially because of the more recent approval of OO,SO‐ILE for pediatric use in the US and lack of experience. Patients receiving OO,SO‐ILE at reduced doses would also be at risk for EFAD if not dosed appropriately. When a patient's LFT results or conjugated bilirubin begin to rise, clinicians may intervene by changing the ILE to a multicomponent ILE formulation to lower the soybean oil content. However, these ILE formulations are lower in essential fatty acids (Table 1) and are dosed differently than SO‐ILE (Table 2). Of note, the multicomponent ILEs also contain less of the downstream metabolites ARA and DHA than FO‐ILE, so the reduced levels of these metabolites in the product may be insufficient to prevent EFAD. Recent literature has suggested that when dosed appropriately, the risk of EFAD is not increased for multicomponent ILE formulations. A retrospective cohort study compared pediatric patients (median age range of 0.23 years to 4.77 years) with intestinal failure receiving SO‐ILE, SO,MCT,OO,FO‐ILE, or FO‐ILE for at least 60 days and found no difference in EFAD (defined as a T:T ratio >0.2) or cholestasis.^85^

**Table 2:** Lipid emulsion and essential fatty acid dosage recommendations including sample calculations of minimal dosing by patient age.

Age	Lipid dose	Amount of fat to prevent EFAD	Example calculations of ILE to prevent EFAD
Pediatrics			
Premature infants	3 g/kg/day SO‐ILE SO,MCT,OO,FO‐ILE OO,SO‐ILE	0.25 g/kg/day LA	1.5 kg × 0.25 g/kg = 0.375 g LA SO‐ILE = 0.375 g LA ÷ 0.108 g LA/ml = 3.47 ml SO,MCT,OO,FO‐ILE = 0.375 g LA ÷ 0.035 LA/ml = 10.7 ml OO,SO‐ILE = 0.375 g LA ÷ 0.0358 LA/ml = 10.47 ml
	1 g/kg/day FO‐ILE		1.5 kg × 1.0 g/kg = 1.5 g fat 1.5 g fat ÷ 0.1 g LA/ml = 15 ml
Term infant (0–12 mo)	2.5–3 g/kg/day SO‐ILE SO,MCT,OO,FO‐ILE OO,SO‐ILE	0.1 g/kg/day LA	3.5 kg × 0.1 g LA = 0.35 g LA SO‐ILE = 0.35 g LA ÷ 0.108 g LA/ml = 3.24 ml SO,MCT,OO,FO‐ILE = 0.35 g LA ÷ 0.035 LA/ml = 10 ml OO,SO‐ILE = 0.35 g LA ÷ 0.0358 LA/ml = 9.77 ml
	1 g/kg/day FO‐ILE		3.5 kg × 1 g/kg = 3.5 g fat 3.5 g ÷ 0.1 g LA/ml = 35 ml
Pediatrics (1–10 y)	2–2.5 g/kg/day SO‐ILE SO,MCT,OO,FO‐ILE OO,SO‐ILE	0.1 g/kg/day LA	23 kg × 0.1 g LA = 2.3 g SO‐ILE = 2.3 g LA ÷ 0.108 g LA/ml = 21.2 ml SO,MCT,OO,FO‐ILE = 2.3 g LA ÷ 0.035 LA/ml = 65.7 ml OO,SO‐ILE = 2.3 g LA ÷ 0.0358 LA/ml = 64.2 ml
	1 g/kg/day FO‐ILE		23 kg × 1.0 g/kg = 23 g fat 23 g ÷ 0.1 g LA/ml = 230 ml
Adults			
Critically ill	<1 g/kg/day SO‐ILE	2%–4% of total energy from LA	70 kg × 30 kcal/kg = 2100 calories 2100 × 3% = 63 calories from LA/day 63 calories ÷ 9 = 7 g LA/day SO‐ILE = 7 g LA ÷ 0.108 g LA/ml = 64.8 ml SO,MCT,OO,FO‐ILE = 7 g LA ÷ 0.035 LA/ml = 200 ml OO,SO‐ILE = 7 g LA ÷ 0.0358 LA/ml = 195 ml
Stable	1 g/kg/day SO‐ILE 1–2 g/kg/day SO,MCT,OO,FO‐ILE 1–1.5 g/kg/day OO,SO‐ILE *All ILE not to exceed 2.5 g/kg/day		

*Note*: Ten percent FO‐ILE is based on 0.1 g fat/ml.^28^ The 20% OO,SO‐ILE mean values used to calculate are 35.8 mg/ml LA and 4.7 mg/ml alpha‐LA.^30^ The 20% SO‐ILE mean values used to calculate are 108 mg/ml LA and 4.5 mg/ml alpha‐LA.^26^ The 20% SO,MCT,OO,FO‐ILE mean values used to calculate are 35 mg/ml LA and 16 mg/ml alpha‐LA.^29^

Abbreviations: EFAD, essential fatty acid deficiency; FO, fish oil; ILE, lipid injectable emulsion; LA, linoleic acid; MCT, medium‐chain triglyceride; OO, olive oil; SO, soybean oil.

## DOSING

EFAD is rare in healthy adults and children, as the amount of linoleic and alpha‐linolenic acid needed to prevent deficiency is minimal. Additionally, fatty acids can also be mobilized from adipose stores during times of inadequate intake, which can prevent deficiency. However, as previously noted, premature infants and those with malnutrition are at increased risk for EFAD because of a lack of adequate fat stores.^86^ Patients receiving an oral diet or enteral nutrition in combination with PN are at lower risk for EFAD if intestinal absorption is adequate.^87^ The volume of ILE required to prevent EFAD varies based on the fatty acid composition of the specific ILE being administered and any oral/enteral intake providing lipid. Calculations must be made to ensure individual essential fatty acid recommendations are met relative to the ILE being prescribed (Table 2) and any other lipid source used (Table 3).^88^ It is also important to note that the amount of essential fatty acids to prevent deficiency may not be the dose of ILE from an energy standpoint needed to optimize growth in the pediatric population or meet the necessary energy requirements in adult patients.

**Table 3:** Fatty acid content of various oil sources.

Oil source	Linoleic acid, g/100 g of oil	Arachidonic acid, g/100 g of oil	Alpha‐linolenic acid, g/100 g of oil	EPA, g/100 g of oil	DHA, g/100 g of oil
Almond	17.4	–	–	–	–
Avocado	12.5	–	1.0	–	–
Canola	18.6	–	9.1	–	–
Cocoa butter	2.8	–	0.1	–	–
Coconut	1.7	–	<0.1	–	–
Corn	53.5	–	1.2	–	–
Fish (cod liver)	0.9	0.9	0.9	6.9	11.0
Fish (salmon)	1.5	0.7	1.1	13.0	18.2
Flaxseed	14.3	–	53.4	–	–
Grapeseed	69.6	–	0.1	–	–
Olive	9.8	–	0.8	–	–
Safflower (oleic)	12.7	–	0.1	–	–
Safflower (linoleic)	74.6	–	–	–	–
Sesame	41.7	–	0.3	–	–
Soybean	50.4	–	6.8	–	–
Sunflower	65.7	–	–	–	–
Walnut	52.9	–	10.4	–	–

*Note*: All data are adapted from the US Department of Agriculture National Nutrient Database.^88^

Abbreviations: DHA, docohexaenoic acid; EPA, eicosapentaenoic acid.

### Premature neonates, infants, and children

Lipid is critical for growth and development in neonates. Preterm infants are born with low adipose reserves. Prompt initiation of adequate energy and essential fatty acid intake is indicated.^83^, ^89^, ^90^ Premature infants are not able to elongate and desaturate essential fatty acids as efficiently as term infants, further increasing the risk of abnormal fatty acid levels. Cumulative lipid intake in the first month of life is associated with significantly greater cerebellar volume.^91^ Additionally, studies in critically ill neonates and older infants have shown that during the acute phase of illness, free fatty acids are mobilized because of increased lipolysis and used as a fuel source with lipid peroxidation correlating with stress severity.^91^, ^92^

Currently available ILEs do not prevent the postnatal decline of ARA and DHA.^93^ Studies have shown ω‐3–containing ILEs further exacerbate the decline in ARA.^65^, ^66^, ^94^ Reduced levels of ARA and DHA have been associated with late‐onset sepsis and chronic lung disease in premature infants.^89^ In a randomized controlled trial of 78 infants requiring PN for up to 28 days who received either OO,SO‐ILE or SO,MCT,OO,FO‐ILE, evaluated serum polyunsaturated fatty acid levels, growth, and comorbidities, including retinopathy of prematurity, bronchopulmonary dysplasia, necrotizing enterocolitis, patent ductus arteriosus, and sepsis, were evaluated.^65^ No differences in growth or comorbidities were observed between the two groups. EPA and DHA were significantly higher in the SO,MCT,OO,FO‐ILE group. Study results confirmed ARA levels decrease after birth regardless of the ILE formulation used; however, postnatal declines were significantly greater in the SO,MCT,OO,FO‐ILE group.^65^

ILEs are sometimes omitted from PN or restricted owing to concerns for hypertriglyceridemia, increasing the risk of EFAD. EFAD may develop within 2–3 days in premature infants without ILE as part of the PN regimen.^45^, ^95^ Premature infants receiving ILE may develop hypertriglyceridemia because of decreased triglyceride clearance. Hypertriglyceridemia may also occur because of excess glucose provision leading to lipogenesis.^45^ When a patient with decreased adipose stores develops EFAD, this may further increase triglyceride values because of de novo lipogenesis from carbohydrate, which is converted to fatty acids and esterified to triglycerides.^83^ In the setting of both EFAD and hypertriglyceridemia, consider providing the minimum amount of fat to meet essential fatty acid requirements while monitoring triglycerides and growth. ESPGHAN guidelines suggest reduction in ILE dosage if plasma triglycerides exceed 265 mg/dl in infants and 400 mg/dl in older children during infusion.^45^ Frequency of triglyceride monitoring may be 1–2 days after an adjustment in ILE dose and weekly to monthly thereafter if levels are stable. More frequent monitoring may be indicated in patients with malnutrition, sepsis, extremely low birth weight, and those receiving a high glucose load. When the triglyceride level is elevated, it is recommended to lower the amount not to stop the ILE infusion.^45^

Early research on oral feeding revealed that prevention of EFAD in infants required a minimum of 1% of the total energy as linoleic acid with the optimal goal of 4% of total energy.^16^ ESPEN/ESPGHAN/European Society for Pediatric Research/Chinese Society of Parenteral and Enteral Nutrition joint guidelines recommend lipid intake should provide 25%–50% of nonprotein energy in pediatric patients receiving PN.^45^ To prevent EFAD, these guidelines recommend a dose of the ILE to provide a minimum linoleic acid intake of 0.25 g/kg/day in premature infants and ≥0.1 g/kg/day in term infants and children (Table 2).^45^ ASPEN guidelines for PN in preterm infants recommend initiation of SO‐ILE or multicomponent ILE (OO,SO‐ILE or SO,MCT,OO,FO‐ILE) at 1–2 g/kg/day with advancement to 3 g/kg/day to support adequate growth.^46^

FO‐ILE is approved in pediatric patients with PN‐associated cholestasis or IFALD at a dose of 1 g/kg/day according to the package insert.^28^ At this dose, EFAD is not observed, and growth is supported.^96^ However, doses up to 1.5 g/kg/day have been tolerated when additional energy is needed.^97^ Fish oil emulsions are cleared more rapidly from the intravascular space than emulsions containing long‐chain triglycerides derived from soy and are less likely to cause hypertriglyceridemia.^98^, ^99^ There have been case reports of FO‐ILE therapy improving hypertriglyceridemia in pediatric patients.^100^

### Adults

The total ILE dosage for adults receiving SO‐ILE, OO,SO‐ILE, or SO,MCT,OO,FO‐ILE is 1–1.5 g/kg/day with a daily dose not to exceed 2.5 g/kg/day (Table 2).^101^ In adults, EFAD can occur when <1%–2% of total energy from linoleic acid is consumed.^101^ EFAD is rare in the general population but may occur in those with lipid‐free PN or with lipid minimization and without adequate dosing of linoleic and alpha‐linolenic acids. ASPEN recommends providing 2%–4% of total energy as linoleic acid and 0.25%–0.5% as alpha‐linolenic acid.^101^ Biochemical EFAD (T:T ratio >0.2) can occur in 7–10 days with fat restriction in adults who are malnourished.^3^, ^102^ In well‐nourished adults, it may take weeks to months of fat‐free PN to develop EFAD.^103^

Patients receiving long‐term PN, lipid‐free PN, or limited ILE doses are at high risk for development of EFAD. Excessive glucose provision may induce hyperinsulinemia, limiting fatty acid mobilization and further increasing the risk of EFAD.^101^ The recommendation for long‐term home PN is to provide 15%–30% of total energy as lipid.^43^ ESPEN guidelines for adult patients with chronic intestinal failure recommend limiting the dose of SO‐ILE to 1 g/kg/day to prevent IFALD with a minimum of 1 g/kg/week to prevent EFAD.^43^ If requiring >1 g/kg/day, the recommendation is to change to a multicomponent ILE lower in ω‐6 fatty acids and phytosterols.^43^ ASPEN dosing recommendations for adults receiving PN vary based on ILE type and patient status. For SO‐ILE in critically ill patients, they recommend <1 g/kg/day, and, once stable, 1 g/kg/day with doses of 1–2 g/kg/day for SO,MCT,OO,FO‐ILE and 1–1.5 g/kg/day for OO,SO‐ILE.^53^ For all ILE formulations, ASPEN recommends not to exceed a dose of 2.5 g/kg/day in adults (Table 2).^53^

## MEASURING FATTY ACIDS

Fatty acids are found in the body as components of triglycerides, phospholipids, cholesteryl esters, and as free fatty acids, with measured fatty acid levels dependent on the pool assayed.^104^, ^105^ Total plasma and serum fatty acids (those present in both free and bound forms) have been found to be less intensive to analyze and correlate well with dietary intake and tissue content, making them more suitable for clinical monitoring.^105^ Most clinically available assays measure total fatty acid levels including in serum, plasma, and red blood cells.^4^, ^5^, ^106^

When assessing the influence of long‐term dietary intake, fatty acid measurements are similar among the various sample types.^105^, ^107^ However, when considering acute changes, such as changes in dietary supplementation or ILE dosing, the time needed for incorporation and stabilization of fatty acids into the different locations becomes relevant. Plasma fatty acid changes occur within days, but the rate of change varies among individuals.^108^, ^109^ Erythrocyte cell membranes reflect fatty acid changes over weeks to months, and whole blood, via venous or dried blood spots, reflects a combination of plasma and erythrocyte fatty acid levels.^109^, ^110^ Tissue incorporation of fatty acids can take months to years to stabilize.^109^ These timelines for fatty acid changes and stabilization are important to consider when choosing the appropriate source for fatty acid clinical monitoring and the interpretation of results in research studies. Plasma fatty acids are often used clinically to monitor essential fatty acid status and are typically obtained monthly or every few months. For patients on stable ILE dosing, such as home PN patients, whole blood or erythrocyte membrane fatty acid levels may also be a reliable source for monitoring. In the setting of research, the length of time on a given ILE dose or supplement should be considered when determining the meaning of alterations in the fatty acid profile.

The techniques used for measurement are also highly important and influence the reported fatty acid levels. The measurement of fatty acids requires numerous steps that can be conducted in multiple ways, including extraction, derivatization, separation, and detection.^111^, ^112^ With variation in the techniques, even when analyzing the same standardized sample, the discrepancy in reported fatty acids is large and can make comparison of reported fatty acid levels between laboratories difficult in both the clinical and research settings.^111^ Variations in analytical techniques are thought to account for at least a portion of variability in reported reference values in healthy populations over time (Table 4).^113^

**Table 4:** Plasma reference ranges from multiple commercially available plasma fatty acid panels by age group.^4^, ^5^

	<1 month		1 month–1 year		1 year–17 years	>1 year	>17 years
Fatty acid	Kish‐Trier et al.^4^	Lagerstedt et al.^5^	Kish‐Trier et al.^4^	Lagerstedt et al.^5^	Lagerstedt et al.^5^	Kish‐Trier et al.^4^	Lagerstedt et al.^5^
LA, nmol/ml	380–3000	350–2660	1240–3890	1000–3300	1600–3500	1210–4300	2270–3850
ALA, nmol/ml	5–150	10–190	20–200	10–190	20–120	20–200	50–130
ARA, nmol/ml	340–1090	110–1110	340–1090	110–1110	350–1030	310–1420	520–1490
EPA, nmol/ml	5–90	2–60	5–90	2–60	8–90	8–130	14–100
DHA, nmol/ml	75–350	10–220	75–350	10–220	30–160	45–365	30–250
Mead, nmol/ml	3–50	8–60	1–32	3–24	7–30	1–35	7–30
T:T ratio	0.006–0.052	0.017–0.083	0.002–0.046	0.013–0.05	0.013–0.05	0.004–0.051	0.010–0.038

Abbreviations: ALA, alpha‐linolenic acid; ARA, arachidonic acid; DHA, docohexaenoic acid; EPA, eicosapentaenoic acid; LA, linoleic acid; T:T ratio, triene to tetraene ratio.

## DIAGNOSING EFAD

### Fatty acid evaluation

Biochemical changes are the first evidence when EFAD develops, occurring within days to weeks of insufficient essential fatty acid dosing, dependent on the age and nutrition status of the patient.^31^, ^103^ When insufficient essential fatty acids, particularly linoleic acid, are provided, increasing levels of unsaturated nonessential fatty acids, including ω‐9 fatty acids, are noted because of substrate availability. This alteration in fatty acid levels leads to an increase in Mead acid and a decrease in ARA levels (Figure 1). The T:T ratio, also known as the Holman index, has historically been used to diagnose EFAD. Recent descriptions of fatty acid levels in healthy populations have reported lower baseline T:T ratios compared with historical reports with reference values varying by age (Table 4).^4^, ^5^, ^106^ This difference in measured fatty acid levels in a healthy reference population has led some groups to raise concern for possible EFAD when the T:T ratio is outside of a given laboratory's normal values.^38^ It is important to note that these reference ranges reflect a specific, limited population and may not reflect “normal” values representative of a larger population. Others have suggested a modified method to interpret fatty acid results by reviewing all fatty acid levels, including linoleic acid, alpha‐linolenic acid, ARA, DHA, EPA, and Mead acid, while considering the lipid source. Of note, the package inserts for OO,SO‐ILE, SO,MCT,OO,FO‐ILE, and FO‐ILE recommend monitoring patients for laboratory evidence and clinical symptoms of EFAD, noting that the T:T ratio may not be adequate to diagnose EFAD and to consider evaluation of fatty acid levels. These approaches, which consider the comprehensive analysis of fatty acids, have been proposed as the T:T ratio alone would not account for the ILE formulation's composition differences, use of altered lipid dosing, and alterations in ω‐3 fatty acid levels.^74^, ^85^, ^86^ Gramlich and colleagues illustrated this point when evaluating the fatty acid profiles of three patients receiving OO,SO‐ILE. They noted a combination of low linoleic acid and ARA levels and elevated Mead acid levels with two patients having elevated T:T ratios.^34^ In the end, it was felt the altered T:T ratio and Mead acid levels were due to the increased oleic acid levels from olive oil with synthesis of Mead acid and not true EFAD, as the ARA levels were within normal limits. The authors suggest that for biochemical EFAD there should be both an increase in the Mead acid level and a decrease in ARA. Changes in only one part of the ratio likely reflect lipid composition and not EFAD. This point is illustrated using patient examples in Table 5. Even though each patient is receiving an adequate dose of ILE to prevent EFAD, the fatty acid panel varies based on ILE content and dosage. Additional concerns have been raised in the setting of FO‐ILEs with increased ω‐3 fatty acid content. With these lipids (SO,MCT,OO,FO‐ILE and FO‐ILE) concern about the possibility of a false negative or falsely normal T:T ratio has been suggested.^84^ With higher EPA and DHA levels, desaturation of oleic acid and production of Mead acid may be limited. This further highlights the importance of reviewing all fatty acid levels when evaluating concern for possible EFAD.

**Table 5:** Example patient fatty acid panels on a variety of lipid emulsions.

Lipid emulsion	SO‐ILE	SO,MCT,OO,FO‐ILE	OO,SO‐ILE^34^, ^114^	FO‐ILE
Patient age, y	0.5	6	45	0.2
Lipid dose, g/kg/day	1.5	1.5	0.5	1
Fatty acid				
Linoleic acid (nmol/ml)	2334	1559	1949	678
Alpha‐linolenic acid (nmol/ml)	97	48	54	61
ARA (nmol/ml)	601	332	621	446
EPA (nmol/ml)	24	230	30	1475
DHA (nmol/ml)	65	254	85	1416
Mead acid (nmol/ml)	7	6	23	5
T:T ratio	0.012	0.018	0.037	0.011

*Note*: All lipid profiles have normal T:T ratios but variable fatty acid levels, reflective of the various ILE product fatty acid contents. All examples are hypothetical patients based on the clinical experience of the authors in addition to literature references.^34^, ^114^

Abbreviations: ARA, arachidonic acid; DHA, docohexaenoic acid; EPA, eicosapentaenoic acid; FO, fish oil; ILE, lipid injectable emulsion; MCT, medium‐chain triglyceride; OO, olive oil; SO, soybean oil; T:T ratio, triene to tetraene ratio.

Depending on the population and fatty acid measured, altered levels may be variably concerning. For example, low DHA or ARA in the preterm neonate may be worrisome, given the importance of these fatty acids in brain and retinal development. In addition, alterations in DHA and ARA levels have been associated with increased risk for neonatal morbidities, including chronic lung disease and late‐onset sepsis, as noted previously.^89^ Additional at‐risk populations to consider include the malnourished, specifically those with protein‐energy malnutrition. These patients often have abnormal fatty acid levels at baseline, including low linoleic acid, elevated Mead acid, and low ARA.^115^ In one study of malnourished children, it was noted that despite 14 days of lipid supplementation, linoleic acid levels improved but ARA levels decreased, further leading to an increase in the T:T ratio. This finding led to the concern that in malnutrition in particular, the T:T ratio may not be the optimal marker to trend EFAD, especially early in treatment.^115^ Other important points to consider when evaluating fatty acid panel results are noted in Table 6.

**Table 6:** Points of consideration when evaluating measured fatty acid levels and possible treatment strategies.

Point of consideration	Treatment strategies
1. Measured fatty acid level	– Source of sample (eg, whole blood, plasma, erythrocyte, or tissue) o Each source affected on different time scale by lipid supplement – All fatty acid levels compared with laboratory reference values (in addition to T:T ratio) – Trend of fatty acid levels over time – Patient state when sample obtained (eg, fasting or ILE infusion or held) o Continuous ILE or postprandial state can falsely alter plasma levels, but erythrocyte is not affected^104^
2. Lipid dosing and source	– Enteral vs parenteral source, including ILE composition o Fatty acid sources can influence the fatty acid panel results (eg, is the patient receiving OO,SO‐ILE with high oleic acid content, and is the patient found to have elevated oleic acid and Mead acid levels but normal T:T ratio) – Dose of ILE ordered vs dose received (eg, stopping for other medication infusions/intravenous incompatibility) o Patient may be receiving inadequate dose if receiving less than prescribed, altering infusion strategies by adding additional access, or changing lipid source or medications could be considerations – Changes in ILE dosing (eg, how recent and adherence) o Plasma fatty acid levels take, on average, 7–10 days of adequate linoleic acid dosing to correct the T:T ratio (other fatty acid sources would take longer)^103^
3. Patient risk factors influencing fatty acid needs or lipid absorption	– Patient age (eg, neonates and ARA and DHA levels) – Pancreatic insufficiency and enzyme dose (ie, can the patient absorb enteral lipid) – Enterally supported patient intake and absorption o Can the patient truly be supported enterally or do they need an additional parenteral source
4. Other concerns or considerations that may relate to fatty acid status	– Other laboratory test result abnormalities (LFT results, triglycerides/lipids, platelet level/function) – Liver disease – Growth in neonatal and pediatric patients – Poor wound healing – Infection concerns o Presence of any of the above findings in addition to altered fatty acid levels, even if the T:T ratio is not at threshold, may influence the clinician to consider increasing fatty acid dosing

Abbreviations: ARA, arachidonic acid; DHA, docohexaenoic acid; ILE, lipid injectable emulsion; LFT, liver function test; OO, olive oil; SO, soybean oil; T:T ratio, triene:tetraene ratio.

### Clinical evaluation

The first human presentations of EFAD were physical in nature because essential fatty acid profiles were not usually assessed before the observable signs and symptoms. EFAD primarily manifests as a dry, scaly rash and weight loss. In children, growth restriction is also common.^13^, ^35^, ^116^ Other physical signs and symptoms of EFAD include alopecia, brittle nails, desquamating dermatitis, increased susceptibility to infection, hair depigmentation, and poor wound healing.^13^, ^21^, ^35^ The presentation of physical manifestations has usually been associated with a T:T ratio >0.4 whereas the biochemical manifestations have been associated with a T:T ratio >0.2.^34^ Besides the elevated T:T ratio, patients with biochemical EFAD have been observed to have elevated LFT results, hyperlipidemia, thrombocytopenia, and altered platelet aggregation.^35^, ^117^

## MONITORING FOR EFAD

No established guidelines for essential fatty acid level monitoring exist, although multiple groups have made recommendations, primarily for pediatric patients. An expert consensus statement from the international summit “Lipids in Parenteral Nutrition” recommended fatty acid profile monitoring in pediatric patients if there is a specific clinical question or concern.^56^ The ASPEN PN safety committee stated in a 2021 consensus recommendation to monitor essential fatty acids in neonatal or pediatric patients if they are malnourished, have signs or symptoms of EFAD, or are receiving lipid minimization dosing of any ILE formulation or in those receiving SO‐ILE <1 g/kg/day.^118^ In the ASPEN ILE recommendations in adult patients, monitoring for EFAD was not mentioned.^53^ The frequency of fatty acid monitoring should depend on patient risk factors, patient stability, and prior testing. The body fluid used for fatty acid testing is important to consider with repeat fatty acid monitoring and changes in the diet or ILE supplementation. Those patients undergoing ILE titration, at increased risk for EFAD, including those receiving decreased ILE doses or with prior EFAD or abnormal fatty acid levels, should have more frequent monitoring with plasma total fatty acids on the order of weeks to months. For those patients on stable ILE dosing and with previously reassuring levels, fatty acid monitoring on the order of months with either plasma, whole blood, or erythrocyte total fatty acid levels can be considered. A nutrition‐focused physical examination, specifically assessing for new onset malnutrition or changes in body composition, in addition to changes in skin and hair, can be helpful to monitor high‐risk patients for signs of EFAD between laboratory monitoring.

## TREATMENT OF EFAD

The threshold for treatment of EFAD is dependent on several factors but most importantly the etiology of the underlying fatty acid abnormalities and how they may influence the patient (Table 6). For example, a lower threshold should be considered for increasing fatty acid dosing in preterm infants, if their levels are abnormal, compared with adult patients. This is because preterm infants have smaller lipid and fatty acid stores as well as the vital importance of fatty acids for their rapid growth and development. This concern for increased risk in neonates is what has led some to consider a lower T:T ratio as threshold for treatment.^38^, ^51^

When possible, the first step to treat EFAD should be to increase fat delivery either through enteral dietary intake (Table 3) or ILE dosing, dependent on the patient's clinical status. In parenterally supported patients, ILE dosage increase has been shown to correct both biochemical and physical manifestations of EFAD if an adequate dose is delivered.^103^, ^119^ It is important to note the biochemical findings typically resolve before the physical findings.^103^ Topical oil therapy has also been attempted in multiple studies to treat both biochemical and physical manifestations of EFAD.^120^, ^121^, ^122^, ^123^, ^124^ However, the results of these studies are mixed, with some showing a difference in both biochemical and physical EFAD with topical oil use and others noting ongoing or worsening of EFAD with a wide range of doses used from approximately 2 mg/kg/day to 1900 mg/kg/day of linoleic acid.^35^, ^120^, ^123^, ^125^, ^126^ These discrepancies make topical oil therapy for EFAD an unreliable strategy and should not be considered if other strategies for increased lipid dosing enterally or parenterally exist. However, in complex patients with EFAD and no other available options to increase essential fatty acid delivery, topical oil therapy could be considered. Of note, further information is needed about the systemic effects of topical oil usage as studies in murine models have shown alteration in circulating cytokine levels with its usage.^127^ The choice of oil used for enteral or topical usage will influence the dose needed to supply an appropriate essential fatty acid dose (Table 3).^88^

Other treatment considerations include cycling PN, decreasing carbohydrate delivery, or giving hypocaloric feeds in the well‐nourished patient to allow for mobilization of fat stores as a source of essential fatty acids.^31^ Use of these strategies should be considered on an individual patient basis and as a possible adjunct to increased essential fatty acid dosing. Historically, whole blood or plasma transfusions were used as treatment strategies for EFAD. These strategies, however, were found to be ineffective given the large volume needed to provide adequate essential fatty acid levels and should not be considered today.^128^, ^129^

## FUTURE CONSIDERATIONS

Laboratory values such as the T:T ratio and free fatty acid levels should be evaluated in the context of the patient clinical scenario to determine the association between fatty acid levels and clinical outcomes. Studies to describe these correlations will enhance our understanding of appropriate fatty acid levels in different patient populations and clinical scenarios, which is imperative, given the widespread use of multicomponent ILEs and their variable fatty acid and oil content. Additionally, consideration should be made to further standardize the techniques used for fatty acid analysis and reporting to make comparisons reliable.

## AUTHOR CONTRIBUTIONS

All authors contributed to the conception, organization, and writing of the manuscript. All authors critically revised the article, agree to be fully accountable for ensuring the integrity and accuracy of the work, and read and approved the final manuscript.

## CONFLICT OF INTEREST STATEMENT

Jodi Wolff is an employee of Baxter Healthcare. Mary Petrea Cober is a consultant for BBraun/CAPS, Baxter, Fresenius Kabi, and Wolters Kluwer. Katie A. Huff was previously a consultant for Baxter Healthcare.
